# Use of the Dyskinesia Impairment Scale in non‐ambulatory dyskinetic cerebral palsy

**DOI:** 10.1111/dmcn.14415

**Published:** 2019-11-29

**Authors:** Helga Haberfehlner, Laura A Bonouvrié, Karin Boeschoten, Sabine Fleuren, Elegast Monbaliu, Jules G Becher, R Jeroen Vermeulen, Annemieke I Buizer

**Affiliations:** ^1^ Department of Rehabilitation Medicine Amsterdam UMC Vrije Universiteit Amsterdam Amsterdam Movement Sciences Amsterdam the Netherlands; ^2^ Department of Neurology Section of Pediatric Neurology Maastricht UMC+ Maastricht the Netherlands; ^3^ Department of Rehabilitation Sciences KU Leuven Campus Brugge Brugge Belgium

## Abstract

**Aim:**

To assess the responsiveness, concurrent validity, and feasibility of the Dyskinesia Impairment Scale (DIS) in non‐ambulatory patients with dyskinetic cerebral palsy (CP).

**Method:**

The study is a secondary analysis of data collected in the IDYS trial, a randomized controlled trial on the effects of intrathecal baclofen (ITB). The DIS and Barry–Albright Dystonia Scale (BADS) were conducted at baseline and after 3 months of ITB or placebo treatment. Responsiveness was assessed by comparing the effect sizes and correlation of change after treatment between the DIS and BADS. Concurrent validity was evaluated by assessing the correlations between scales. Feasibility was evaluated for each DIS item by the number of participants who successfully accomplished the item.

**Results:**

Thirty‐three non‐ambulatory patients (9 females, 24 males) with dyskinetic CP (ITB‐treated: *n*=17, mean [SD] age: 14y 1mo [4y 1mo]; placebo‐treated: *n*=16, mean [SD] age: 14y 7mo [4y]) were included in the study. The effect sizes for BADS and DIS were similar in The ITB‐treated group (−0.29 and −0.22 respectively). Changes after treatment on the DIS dystonia subscale correlated with changes on the BADS (*r*=0.64; *p*<0.001). The DIS dystonia subscale and BADS correlated at baseline and follow‐up (*r*=0.78; *p*<0.001 and *r*=0.79; *p*<0.001). Not all DIS activity items could be performed in this sample of patients.

**Interpretation:**

For non‐ambulatory patients with dyskinetic CP, the responsiveness of the DIS equalled the responsiveness of BADS. Concurrent validity was adequate. Feasibility for activity items was restricted in patients with severe dyskinetic CP.

**What this paper adds:**

- The Dyskinesia Impairment Scale (DIS) and Barry–Albright Dystonia Scale showed similar responsiveness in non‐ambulatory patients with dyskinetic cerebral palsy (CP).
- No floor or ceiling effect was observed for DIS in non‐ambulatory participants.
- The concurrent validity of DIS was adequate in non‐ambulatory participants.
- Patients with dyskinetic CP in Gross Motor Function Classification System levels IV and V could not perform all DIS activity items.

## What this paper adds

- The Dyskinesia Impairment Scale (DIS) and Barry–Albright Dystonia Scale showed similar responsiveness in non‐ambulatory patients with dyskinetic cerebral palsy (CP).
- No floor or ceiling effect was observed for DIS in non‐ambulatory participants.
- The concurrent validity of DIS was adequate in non‐ambulatory participants.
- Patients with dyskinetic CP in Gross Motor Function Classification System levels IV and V could not perform all DIS activity items.

**Abbreviations**
BADS: Barry–Albright Dystonia Scale
DIS: Dyskinesia Impairment Scale
ITB: Intrathecal baclofen

The prevalence of cerebral palsy (CP) is estimated from 1.5 to more than 4 in every 1000 live births worldwide, and is thereby the most common physically disabling condition in childhood.^1^ Dyskinetic CP accounts for 6% to 15% of all children with CP and is the most disabling form of CP.^2^ Children with dyskinetic CP experience limitations in mobility, manual ability, and communication due to involuntary movements. These dyskinetic movements are characterized by two features, which often coexist in the same patient: (1) dystonia, described as abnormal patterns of posture due to sustained muscle contractions; and (2) choreoathetosis, characterized by faster involuntary, uncontrolled, recurring, and occasionally stereotyped movements.^2^, ^3^

In the last decade, promising treatment options have become available for patients with dyskinetic CP.^3^ Treatments such as intrathecal baclofen (ITB) aim to minimize the dyskinetic movements that underlie the functional limitations in children and young adults with dyskinetic CP. Several scales to measure dystonia have been described in the literature.^4^ The Barry–Albright Dystonia Scale (BADS)^5^ is the most commonly used scale to evaluate treatment outcome in children with dyskinetic CP.^6^, ^7^, ^8^, ^9^ More recently, the Dyskinesia Impairment Scale (DIS) was developed by Monbaliu et al.^10^ The DIS has a more comprehensive content compared to the BADS, including a detailed video protocol with predefined activities and positions used to score dyskinetic movements.^4^, ^10^ The DIS thoroughly evaluates both dystonia and choreoathetosis during rest and activity, and takes into account the duration and amplitude of dystonia and choreoathetosis.^4^, ^10^ Currently, there are no other scales assessing choreoathetosis.^4^ No reports on responsiveness to change are available^4^ and concurrent validity was established by only one study.^10^

The primary aim of the present study was to assess the responsiveness of the DIS. The secondary aim was to assess concurrent validity and report on the feasibility of using the DIS in a group of children and young adults with dyskinetic CP selected for ITB treatment.

We assumed that a group treated with ITB would show a decrease in the measured constructs (i.e. dyskinetic movements), while the control group treated with placebo would show no change in the measured constructs. Our hypotheses were: (1) effect sizes would be higher for the DIS in a group treated with ITB compared to a control group treated with placebo; (2) effect sizes would be higher for the DIS dystonia subscale compared to the BADS; and (3) change after treatment (ITB or placebo) on the DIS dystonia subscale would correlate with the change on the BADS.

## Method

The study is a secondary analysis of data collected in a previously reported multicentre, double‐blind, placebo‐controlled trial on the effect of ITB in patients with dyskinetic CP (IDYS trial).^11^, ^12^ The IDYS trial was conducted at the Vrije Universiteit Medical Center (part of Amsterdam University Medical Centers, Amsterdam) and the Maastricht University Medical Center, the Netherlands. The study was approved by the Medical Ethics Committee of the Vrije Universiteit Medical Center, Amsterdam. Written informed consent was obtained from participants and/or their parents.

### Participants

The participant inclusion criteria for the IDYS trial were: (1) presenting with dyskinetic CP; (2) classified in Gross Motor Function Classification System (GMFCS) levels IV and V; (3) aged 4 to 25 years; (4) lesions on magnetic resonance imaging; and (5) eligible for ITB treatment using commonly applied criteria.^11^, ^12^ The exclusion criteria were: (1) contraindication for general anaesthesia or baclofen; (2) oral pharmacological treatment was sufficient; (3) deep brain stimulation; (4) ventriculoperitoneal shunt; and (5) other disorders interfering with treatment. All participants of the IDYS trial who completed the DIS and BADS at baseline and 3 months after pump implantation were included in the current analysis.

### Measurements

The DIS dystonia subscale and BADS were used to assess dystonia.^5^, ^10^ The DIS choreoathetosis subscale was used to assess choreoathetosis.^10^

The DIS evaluates 12 body regions (eyes, mouth, neck, trunk, right and left arm proximal, right and left arm distal, right and left leg proximal, and right and left leg distal). The duration (percentage of time) and amplitude (percentage of range) of dystonia and choreoathetosis were assessed at rest and during activity using a detailed video protocol that defined body position (sitting in a comfort position or active position, lying, and standing) as well as activities the child had to perform (eye tracking, eye blinking, mouth opening/closing, speaking, lateroflexion of the head, rotation of the head, active sitting position, forward flexion of the trunk, arm abduction, grasping and reaching for a pen, grasping and moving a cup, rolling, standing, and heel/toe raising). Duration and amplitude were scored on a 5‐point ordinal scale.^10^

The BADS rates the severity of dystonia on a 5‐point scale for eight body regions (eyes, mouth, neck, trunk, left and right upper extremities, left and right lower extremities).^5^ The BADS has shown responsiveness to change in dystonia after interventions in patients with dyskinetic CP^5^, ^6^, ^8^, ^9^, ^13^ and was therefore considered as the criterion standard in the current study. The BADS is reported to have moderate concurrent validity.^4^, ^14^, ^15^

Brief video sequences of all patients were made, according to the DIS video protocol.^10^ Two experienced paediatric therapists (KB and SF) were trained to score the DIS and BADS and scored all videos. They were blinded to the measurement time points (baseline and follow‐up) and group allocation and scored the videos in random order within a year’s time. The same rater assessed both time points (baseline and follow‐up) and both scales (DIS and BADS) in an individual child. Scoring of the BADS was performed on a selection of videos recorded for the DIS: sitting at rest; speaking (if performed); grasping/reaching for a pen from a lying position (left and right), and rolling over left and right.

A percentage score for all subscales was calculated by dividing the individual score by the maximum possible score on the corresponding item or subscale.^10^ Although all possible items of the DIS were evaluated in each participant and scored at each time point, the final score of the individual participant included only those items performed at both time points. This approach was chosen because the number of performed items varied between time points.

Patient and clinical characteristics included GMFCS^16^ and Manual Ability Classification System levels.^17^ Furthermore, as a measure of cognitive function, comprehension of spoken language was tested with the Computer‐Based instrument for Low Motor Language Testing.^18^

### Responsiveness

Responsiveness is defined ‘as the ability for an instrument to detect change over time in the construct to be measured’.^19^ In the current study, we used the effect sizes of the DIS to evaluate responsiveness. The criteria proposed by Cohen were used for interpretation: an effect size greater than 0.80 was considered large, 0.50 to 0.80 moderate, and 0.20 to 0.50 small.^20^ In our study, a negative effect size meant a favourable effect (decrease in dystonia/choreoathetosis), while a positive effect size meant an unfavourable effect (increase of dystonia/choreoathetosis). Using effect size to evaluate the responsiveness of an instrument has been described as inappropriate without using a comparison instrument measuring the same construct.^21^ Therefore, we compared the effect size of the DIS dystonia subscale to the effect size of the BADS. As an additional measure of responsiveness, we calculated the correlation of the change in scores, defined as the difference between baseline and follow‐up scores, between the DIS dystonia subscale and BADS. To assess floor and ceiling effects, the percentage of patients achieving the worst and best possible score at baseline and follow‐up was assessed for the DIS dystonia subscale, BADS, and DIS choreoathetosis subscale. A percentage of more than 15% was considered as a floor or ceiling effect.^22^, ^23^

### Concurrent validity

To establish the concurrent validity of the DIS, we considered the score of the DIS and the score for the criterion standard (i.e. BADS) at the same time point, that is, at baseline and follow‐up.^21^ We assessed the correlation between the DIS dystonia subscale and BADS at both time points.

### Feasibility

To evaluate how feasible it is to perform the DIS in non‐ambulatory children and young adults, for each item we assessed the number of participants who could successfully accomplish the item at both time points. An activity was considered to be successfully accomplished, and consequently scored, when at least an intention of the movement during the requested activity was seen, whereas a rest item was scored when the body position (i.e. sitting in a comfort position and lying down) could be achieved.

### Statistical analysis

Effect size was calculated as the mean change between baseline and follow‐up scores divided by the SD of the baseline scores.^21^ For all variables, normality was tested using the Shapiro–Wilk test. Correlations were calculated with Pearson’s correlation coefficient. Statistics were calculated with SPSS 22.0 (IBM Corp., Armonk, NY, USA). A *p*<0.05 was considered statistically significant.

## Results

### Participants

The DIS and BADS scores were available at both time points for 33 (9 females, 24 males) of the 36 participants from the IDYS trial (17 in the ITB‐treated group mean [SD] age: 14y 1mo [4y 1mo] and 16 in the placebo‐treated group; mean [SD] age: 14y 7mo [4y]). Patient and clinical characteristics such as age, sex, weight, height, GMFCS level, Manual Ability Classification System level, and age‐equivalent comprehension of spoken language measured by the Computer‐Based instrument for Low Motor Language Testing are described in Table 1.

**Table 1:** Patient and clinical characteristics of participants at baseline (intrathecal baclofen‐treated group and placebo‐treated control group)

	ITB‐treated group (*n*=17)	Placebo‐treated group (*n*=16)
Mean age (SD); range (y:mo)	14:1 (4:1); 9:4–22:8	14:7 (4:0); 9:0–20:5
Sex		
Female	4 (24)	5 (31)
Male	13 (76)	11 (69)
Mean weight (SD); range (kg)	35.2 (13.3); 20–75	31.9 (9.1); 20–53
Mean height (SD); range (cm)	148.6 (20.9); 118–181	147.8 (19.2); 110–175
GMFCS level		
IV	8 (47)	5 (31)
V	9 (53)	11 (69)
MACS level		
III	2 (12)	1 (6)
IV	4 (23)	4 (25)
V	11 (65)	11 (69)
C‐BiLLT (age‐equivalent of comprehension of spoken language)		
<2y	1 (6)	3 (19)
2–3y	1 (6)	2 (12)
>3y	12 (70)	10 (63)
Missing	3 (18)	1 (6)

Data are *n* (%) unless otherwise stated. ITB, intrathecal baclofen; GMFCS, Gross Motor Function Classification System; MACS, Manual Ability Classification System; C‐BiLLT, Computer‐Based instrument for Low Motor Language Testing.

### Measurements

The mean (SD) follow‐up time was 3.0 (0.2) months in the ITB‐treated group and at 2.8 (0.6) months in the placebo‐treated group. A decrease as well as an increase in performed items of the DIS occurred between baseline and follow‐up, ranging between −8 and +10 items. Performed items did not differ between the ITB‐ and placebo‐treated groups (*p*=0.312).

### Responsiveness

**Figure 1:**
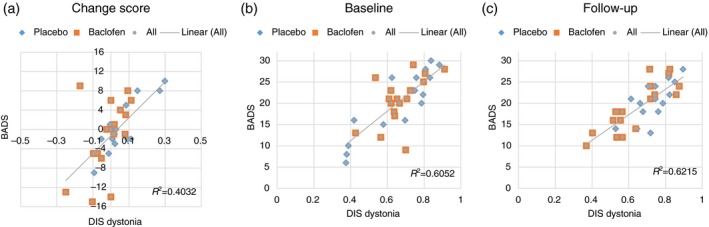
(a) Change in score between baseline and follow‐up for the intrathecal baclofen‐treated and control groups (treated with placebo). (b) Correlation between the Dyskinesia Impairment Scale (DIS) dystonia subscale and Barry–Albright Dystonia Scale (BADS) at baseline and (c) at follow‐up. [Color figure can be viewed at http://wileyonlinelibrary.com]

Table S1 (online supporting information) shows the effect sizes for the DIS and BADS scores for all body regions, aspects, and subscales per group. In the ITB‐treated group, the effect size for the DIS dystonia subscale was −0.22 (small favourable effect), while in the placebo‐treated group the effect size was +0.34 (small unfavourable effect). The effect size of the BADS was −0.29 (small favourable effect) in the ITB‐treated group and 0.04 in the placebo‐treated group (no effect). The effect size of the BADS in the ITB‐treated group was comparable to the effect size for the DIS dystonia subscale. However, for the placebo‐treated group the effect size on the DIS dystonia subscale showed a small unfavourable effect, which was not measured on the BADS. The correlation of change scores between the DIS dystonia subscale and BADS was 0.64 (95% confidence interval (CI) 0.37–0.80; *p*<0.001) (Fig. 1a).

The effect size in the ITB‐treated group for the total DIS choreoathetosis subscale was −0.25 (small favourable effect). In the placebo‐treated group, the effect size was +0.10 (no effect) (Table S1).

None of the participants achieved the worst and/or best possible score for the DIS dystonia subscale or BADS, meaning that no floor or ceiling effect occurred in our patient population for these outcome measures. For the choreoathetosis subscale of the DIS, 4 out of 33 participants (12%) scored the best possible score at baseline and 3 (9%) scored the best possible score at follow‐up. None of the participants achieved the worst score at either of the time points. Therefore, the choreoathetosis subscale also showed no floor or ceiling effect in our patient population.

### Concurrent validity

The Pearson’s correlation coefficients between the DIS dystonia subscale and BADS at baseline and follow‐up were 0.78 (95% CI 0.59–0.88; *p*<0.001) and 0.79 (95% CI 0.60–0.89; *p*<0.001) respectively (Fig. 1b,c).

### Feasibility

No item of the video protocol used to assess dystonia and choreoathetosis during activity could be performed by all participants. The number of participants who successfully performed the requested activity items ranged from 0 to 29 out of the 33 participants (Table 2). Only 12 out of 33 participants could successfully perform the item ‘speech’; the others were non‐speaking children. In our patient population, no participant could accomplish the items of the DIS that asked for a ‘sitting‐active position’ and ‘standing position’. Therefore, the body region ‘trunk’ was not included in the evaluation of dystonia and choreoathetosis during activity in our patient population. The resting body positions could be achieved by all but one child. In this child, lying was not possible due to pain at the hip at the 3‐month follow‐up.

**Table 2:** Percentage of participants that are able to perform requested activities of the Dyskinesia Impairment Scale (DIS) to assess dystonia and choreoathetosis

Body region	Activity	Position	Participants able to perform the item^a^ (*n*=33)	%
Eyes	Eye tracking	Sitting (comfort position)	29	88
	Eye blinking	Sitting (comfort position)	27	82
Mouth	Opening/closing mouth	Sitting (comfort position)	28	85
	Speaking	Sitting (comfort position)	12	36
Neck	Lateroflexion of the head	Sitting (comfort position)	24	73
	Rotation of the head	Sitting (comfort position)	29	88
Trunk	Active sitting position	Sitting (active position)	0	0
	Forward flexion	Sitting (active position)	0	0
Arm proximal right/left	Arm abduction	Sitting (comfort position)	28	85
	Grasping/reaching for a pen	Lying position	28	85
Arm distal right/left	Grasping and moving a cup^a^	Sitting (comfort position)	28	85
	Grasping and moving a pen^a^	Sitting (comfort position)	29	88
Leg proximal right/left	Rolling	Lying position	26	79
	Standing	Standing position	0	0
Leg distal right/left	Rolling	Lying position	24	73
	Heel/toe raise	Sitting (comfort position)	25	76

a If at least the intention of movement was visible, the activity was scored as the participant being able to perform it.

## Discussion

This study reports on the responsiveness, concurrent validity, and feasibility of the DIS in a group of non‐ambulatory children and young adults (in GMFCS levels IV and V), treated with ITB compared to placebo.

### Responsiveness

As expected, the effect sizes for the DIS dystonia subscales (active, resting, and total) and DIS choreoathetosis subscales (active, resting, and total) showed a more positive effect in the ITB‐treated group compared to the placebo‐treated group (ITB: −0.31 to −0.18; placebo: −0.04 to +0.44), and changes in the DIS dystonia subscale correlated with changes on the BADS. However, overall effect sizes in the ITB‐treated group were small.

Contrary to our hypothesis, the effect sizes of the DIS dystonia subscale (ITB: −0.22; placebo group: +0.34) did not exceed the effect sizes on the BADS (ITB: −0.29; placebo: +0.04). Compared to the literature, the measured effect sizes on the BADS were relatively low in the current study. Barry et al.^5^ showed an effect size of −2.3 on the BADS with ITB treatment in secondary dystonia due to CP or acquired brain injury (age range 3–42y). In addition, another study in a group of children with dystonia due to various aetiologies treated with intraventricular baclofen (age 2–28y) carried out by the same research group also showed a larger effect size of the BADS (−2.2) than recorded in the current study.^13^ However, the major drawbacks of these studies included the rater of the BADS not being blinded and the absence of a control group. Furthermore, the evaluation of BADS was performed by the same group who developed the test, which may have biased the results.^5^ These drawbacks might explain the discrepancy with our results.

We acknowledge that in our study both DIS and BADS may not have captured all improvements that occurred in dystonia and choreoathetosis due to ITB treatment. This is because the measured effect on the primary outcome measure of the IDYS trial – goal attainment scaling – was much larger than the effect measured by the DIS.^12^ We kept the circumstances where the DIS and BADS were performed at baseline and follow‐up as constant as possible (i.e. tester, testing room, time of day). However, at follow‐up, after the DIS and BADS were performed, patients and parents were told about group allocation (ITB‐ or placebo‐treated). This may have led to anticipation, with excitement or stress, which may have influenced the severity of dystonia and choreoathetosis on the day, since stimuli such as emotion, stress, and pain are known to exacerbate dystonia.^3^ We used the BADS as the criterion standard to assess responsiveness. It might be questioned if the BADS can be used as the criterion standard, since responsiveness has been mainly assessed in studies not designed to evaluate responsiveness and without prior knowledge of the magnitude of improvement caused by the intervention.^4^, ^5^, ^6^, ^8^, ^9^, ^13^ Due to these limitations of the BADS, in the current study we used effect sizes with a prior hypothesis as well as the correlation in change scores between the DIS dystonia subscale and BADS. We deem this approach appropriate to draw conclusions on the responsiveness of the DIS for the tested group of children and young adults compared to the BADS.

In summary, the responsiveness of the DIS dystonia subscale equals the responsiveness of the BADS. The DIS choreoathetosis subscale showed a similar effect size; however, no comparison scale is available for the assessment of choreoathetosis.

### Concurrent validity

Concerning concurrent validity of the DIS dystonia subscale, a good correlation with the BADS was found in our selected group at baseline and follow‐up. Results were comparable to the results of previous research.^10^

### Feasibility

The feasibility of the DIS activity items in a population of children and young adults with dyskinetic CP selected for ITB treatment (GMFCS levels IV and V; Manual Ability Classification System levels III–V) was restricted due to the inability of this group of patients to perform all activities from the DIS video protocol used to assess dystonia and choreoathetosis. There were several underlying reasons. First, impaired gross motor function did not allow activities such as active sitting and standing, making evaluation of dystonia of the trunk during activity impossible and limited evaluation of dystonia in the proximal leg during activity. Second, a large number of patients were non‐speaking, making evaluation of dystonia during speech impossible. Third, some patients did not show any intention of movement for several items that were performed in a supported sitting or lying position. This might be due to impaired gross motor function, manual ability, oral motor dysfunction, or low comprehension of the assignment. That low comprehension of spoken language has affected performance on the DIS in some group members is possible, but it probably did not play a major part since only 1 of 17 children in the ITB group and 3 of 16 children in the placebo group scored an age‐equivalent of younger than 2 years on the Computer‐Based instrument for Low Motor Language Testing (Table 1). Note that since approximately 80% of children with dyskinetic CP are classified as in GMFCS levels IV and V,^24^ generally a large group of children with dyskinetic CP will not be able to perform all activity items of the DIS.

When the feasibility of the full DIS protocol is compared to the BADS, we conclude that all items of the BADS could be scored in all participants of our study; however, this is mainly because the activities or positions to score dystonia are not as precisely defined for the BADS.

### Implications and future perspectives

The DIS dystonia subscale and BADS show equal responsiveness. Yet, considering that the DIS is time‐consuming, the advantage of the DIS in scoring both dystonia and choreoathetosis over the BADS, and its utility in clinical practice, need to be critically appraised. Presently, the DIS shows no advantage concerning responsiveness for assessing dystonia in patients with dyskinetic CP within an ITB clinical trial for non‐ambulatory children. Nevertheless, in addition to dystonia, the DIS also assesses choreoathetosis. At the individual level, the generally more comprehensive content of the DIS, compared to the BADS, might be an advantage. Test–retest reliability, including measurement error (i.e. standard error of measurement and smallest detectable change), should be used to determine if individual changes are due to treatment and not to measurement error.^25^ Feasibility of the full DIS protocol is limited because many activities items of the DIS could not be performed in non‐ambulatory children. Future research should elaborate the activity part of the DIS scale towards a reduction of activity items for this group of patients. Another option would be to explore whether in a severely impaired group of participants the dystonia and choreoathetosis at rest (in the way it is assessed in the DIS) would sufficiently cover the scoring of dyskinetic movements.

## Conclusion

The DIS dystonia subscale shows similar responsiveness in comparison to the BADS in non‐ambulatory children and young adults with dyskinetic CP. Concurrent validity of the DIS is adequate as reported previously. Feasibility of the DIS activity items is restricted in patients with dyskinetic CP in GMFCS levels IV and V. Reducing the activity items of the DIS should be investigated.

## Supporting information


**Table S1**: Effect sizes of the DIS and BADS for all body regions and aspects per group.
