# Supplementary material for: Use of the Dyskinesia Impairment Scale in non‐ambulatory dyskinetic cerebral palsy
Source: Dev Med Child Neurol. 2019 Nov 29;62(4):494–9. doi: 10.1111/dmcn.14415 (PMC7079134; doi:10.1111/dmcn.14415)
Supplement: Supplementary file 1 — Table S1: Effect sizes of the DIS and BADS for all body regions and aspects per group. [file DMCN-62-494-s001.docx]

**Table S1**: Effect sizes of the Dyskinesia Impairment Scale (DIS) and Barry–Albright Dystonia Scale (BADS) for all body regions and aspects per group. A negative effect size corresponds to a favourable effect since a decrease in dystonia and choreoathetosis is expressed by a lower score, while a positive effect size means an unfavourable effect

| **DIS** | **Active** | | | | | | **Rest** | | | | | | **Total** | | **BADS** |  |  |
| --- | --- | --- | --- | --- | --- | --- | --- | --- | --- | --- | --- | --- | --- | --- | --- | --- | --- |
|  | Duration | | Amplitude | | Total | | Duration | | Amplitude | | Total | | ∑^a^ | ∑ |  |  |  |
| Group | Control | ITB | Control | ITB | Control | ITB | Control | ITB | Control | ITB | Control | ITB | Control | ITB |  | Control | ITB |
| **Dystonia** | | | | | | | | | | | | | | | | | |
| Eyes | 0.52 | 0.00 | 0.61 | 0.12 | 0.49 | 0.00 | 1.09 | 0.23 | 0.45 | 0.04 | 0.70 | 0.14 | 0.70 | 0.07 | Eyes | 0.25 | −0.08 |
| Mouth | 0.12 | −0.21 | −0.14 | −0.20 | −0.20 | −0.21 | −0.10 | 0.00 | −0.22 | −0.13 | −0.18 | −0.08 | −0.15 | −0.21 | Mouth | −0.17 | 0.21 |
| Neck | 0.03 | 0.04 | −0.07 | −0.51 | 0.03 | 0.04 | 0.33 | 0.20 | 0.72 | 0.00 | 0.49 | 0.14 | 0.26 | 0.08 | Neck | −0.04 | −0.27 |
| Trunk |  |  |  |  |  |  | 0.37 | 0.67 | 0.39 | 0.06 | 0.39 | 0.41 | 0.39 | 0.43 | Trunk | −0.33 | −0.11 |
| Arm right proximal | 0.05 | −0.18 | 0.03 | −0.16 | 0.05 | −0.18 | 0.26 | −0.04 | 0.12 | −0.26 | 0.20 | −0.16 | 0.17 | −0.18 | Arm right | −0.14 | −0.39 |
| Arm right distal | −0.03 | 0.13 | 0.00 | 0.06 | −0.03 | −0.02 | 0.32 | −0.46 | 0.05 | −0.26 | 0.05 | −0.40 | −0.01 | −0.09 |  |  |  |
| Arm left proximal | 0.22 | −0.26 | 0.35 | 0.08 | 0.22 | −0.26 | 0.00 | −0.34 | 0.08 | 0.08 | 0.05 | −0.15 | 0.20 | −0.19 | Arm left | −0.07 | −0.42 |
| Arm left distal | 0.39 | −0.18 | 0.18 | −0.64 | 0.32 | −0.34 | 0.63 | −0.47 | 0.55 | −0.36 | 0.40 | −0.50 | 0.49 | −0.56 |  |  |  |
| Leg right proximal | 0.00 | 0.11 | −0.22 | −0.06 | −0.10 | 0.07 | 0.43 | −0.19 | 0.00 | −0.38 | 0.16 | −0.32 | 0.20 | −0.17 | Leg right | 0.57 | −0.80 |
| Leg right distal | −0.15 | −0.34 | −0.27 | 0.00 | −0.65 | −0.19 | 0.18 | 0.47 | −0.24 | 0.05 | −0.34 | 0.25 | −0.33 | −0.03 |  |  |  |
| Leg left proximal | −0.21 | 0.10 | −0.17 | 0.22 | −0.28 | 0.17 | 0.25 | −0.29 | −0.22 | −0.27 | −0.04 | −0.31 | −0.05 | −0.06 | Leg left | 0.35 | -0.49 |
| Leg left distal | 0.23 | −0.64 | 0.28 | −0.11 | 0.32 | −0.51 | 0.19 | 0.21 | 0.22 | −0.23 | 0.07 | −0.03 | 0.34 | −0.36 |  |  |  |
| **Dystonia total** | 0.16 | −0.26 | 0.19 | −0.18 | 0.20 | −0.27 | 0.52 | −0.04 | 0.32 | −0.32 | 0.44 | −0.18 | 0.34 | −0.22 | **BADS total** | 0.04 | −0.29 |
| **Choreoathetosis** | | | | | | | | | | | | | | | | | |
| Eyes | 0.30 | −0.25 | 0.24 | −0.40 | 0.42 | −0.25 | 0.00 | −0.35 | −0.19 | −0.36 | −0.14 | −0.33 | 0.04 | −0.28 |  | | |
| Mouth | 0.44 | −0.37 | 0.44 | −0.22 | 0.43 | 0.43 | −0.09 | −0.27 | 0.09 | −0.21 | 0.05 | −0.23 | 0.24 | −0.24 |  |  |  |
| Neck | 0.17 | 0.94 | 0.00 | 1.47 | 0.17 | 0.90 | −0.09 | −0.45 | 0.00 | −0.55 | −0.05 | −0.45 | 0.07 | −0.16 |  |  |  |
| Trunk |  |  |  |  |  |  | −0.10 | 0.00 | 0.00 | −0.18 | −0.09 | 0.00 | −0.09 | 0.00 |  |  |  |
| Arm right proximal | 0.00 | −0.09 | −0.03 | 0.05 | 0.00 | −0.08 | 0.10 | −0.34 | 0.25 | −0.30 | 0.19 | −0.32 | 0.07 | −0.17 |  |  |  |
| Arm right distal | 0.40 | 0.28 | 0.02 | 0.02 | 0.16 | 0.13 | −0.11 | −0.13 | 0.00 | −0.20 | −0.09 | −0.17 | −0.01 | 0.02 |  |  |  |
| Arm left proximal | 0.05 | 0.07 | 0.05 | 0.10 | 0.05 | 0.07 | 0.18 | −0.26 | 0.40 | −0.31 | 0.45 | −0.28 | 0.17 | −0.04 |  |  |  |
| Arm left distal | 0.04 | −0.34 | −0.07 | −0.41 | −0.03 | −0.40 | 0.21 | −0.46 | 0.17 | −0.47 | 0.11 | −0.47 | 0.02 | −0.46 |  |  |  |
| Leg right proximal | 0.13 | 0.39 | 0.46 | 0.10 | 0.27 | 0.28 | −0.19 | −0.11 | −0.20 | −0.05 | −0.20 | −0.09 | 0.03 | 0.09 |  |  |  |
| Leg right distal | 0.11 | −0.31 | 0.26 | −0.38 | 0.20 | −0.37 | −0.23 | −0.12 | 0.00 | −0.08 | 0.00 | −0.10 | 0.15 | −0.28 |  |  |  |
| Leg left proximal | −0.06 | 0.06 | 0.00 | −0.05 | −0.03 | 0.00 | 0.05 | 0.27 | −0.05 | 0.23 | 0.05 | 0.29 | 0.04 | 0.13 |  |  |  |
| Leg left distal | −0.11 | −0.23 | −0.03 | −0.27 | −0.06 | −0.26 | −0.31 | −0.13 | −0.25 | −0.21 | −0.15 | −0.19 | −0.06 | −0.25 |  |  |  |
| **Choreoathetosis total** | 0.29 | −0.16 | 0.26 | −0.28 | 0.28 | −0.23 | −0.06 | −0.30 | −0.01 | −0.31 | −0.04 | −0.31 | 0.10 | −0.25 |  |  |  |

^a^Sum of active and resting aspect of DIS. ITB, intrathecal baclofen.
